# Supplementary material for: Targeting integrin αvβ3 by a rationally designed protein for chronic liver disease treatment
Source: Commun Biol. 2021 Sep 16;4:1087. doi: 10.1038/s42003-021-02611-2 (PMC8445973; doi:10.1038/s42003-021-02611-2)
Supplement: Supplementary file 2 — Supplementary Information [file 42003_2021_2611_MOESM2_ESM.pdf]

Supplementary material

Targeting Integrin $\alpha_v\beta_3$ by A Rationally Designed Protein for Chronic Liver Disease Treatment

Ravi Chakra Turaga[1], Ganesh Satyanarayana[1], Malvika Sharma[1], Jenny J. Yang[2], Shiyuan Wang[3], Chunfeng Liu[3], Sun Li[3], Hua Yang[4], Hans Grossniklaus[4], Alton Brad Farris[5], Jordi Gracia-Sancho[6], and Zhi-Ren Liu[1*]

[1]Department of Biology, [2]Department of chemistry Georgia State University, Atlanta, GA 30303, USA [3]Amoytop Biotech Inc., Xiamen, P. R. China, [4]Department Ophthalmology [5]Department of Pathology Emory University, Atlanta, GA 30322 USA, [6]IDIBAPS Biomedical Research Center & CIBEREHD, Barcelona, Spain

*Corresponding Author:

Zhi-Ren Liu, Ph.D.
Department of
Biology Georgia
State University
University Plaza
Atlanta, GA 30303
USA
(zliu8@gsu.edu)

Supplementary table 1. Antibodies, reagents, and cell lines used in the study

Reagent or Resource	Source	Identifier
Ultravision Peroxidase block	Thermo Scientific	TA060H2O2Q
Ultravision Protein block	Thermo Scientific	TA060PBQ
Antibody diluent OP Quanto	Thermo Scientific	TA-125-ADQ
Betazoid DAB Chromogen kit	Biocare Medical	BDB2004L
DEPEX mounting media	Electron Microscopy Sciences	13514
Coverslips	Thermo Scientific	102420
Tris Base	Fisher Scientific	BP154-1
Tween-20	Sigma-Aldrich	P5927
Citrate buffer	Sigma-Aldrich	C9999-1000ML
Xylenes	Fisher Scientific	X5-4
Large Volume Mayer's Hematoxylin	Thermo Scientific	TA-125-MH
Lipofectamine® RNAiMAX	ThermoScientific	13778030
integrin β3 siRNA (h)	Santa-Cruz Biotechnology	sc-29375
**Antibodies**		
α-SMA (1:500)	Sigma	A2547
cleaved caspase-8 (1:100)	Cell Signaling Technology	9496
PECAM1 (1:1000)	Abcam	Ab32457
SE-1 (1:200)	Novus Biologicals	NB11068095
GAPDH	Santa-Cruz Biotechnology	Sc-47724
Hif1α (1:200)	Millipore	MAB5382
Integrin αV	Cell Signaling Technology	4711
Integrin β3	Millipore	AB2984
**Critical Commercial Assay kits**		
Hydroxyproline Assay	Sigma-Aldrich	MAK-008
Picro Sirus red staining kit	IHC world	IW-3012
annexin V/Dead Cell Apoptosis Kit	ThermoScientific	V13241
**Cell Lines**		
LX-2	EMD Millipore	SCC064

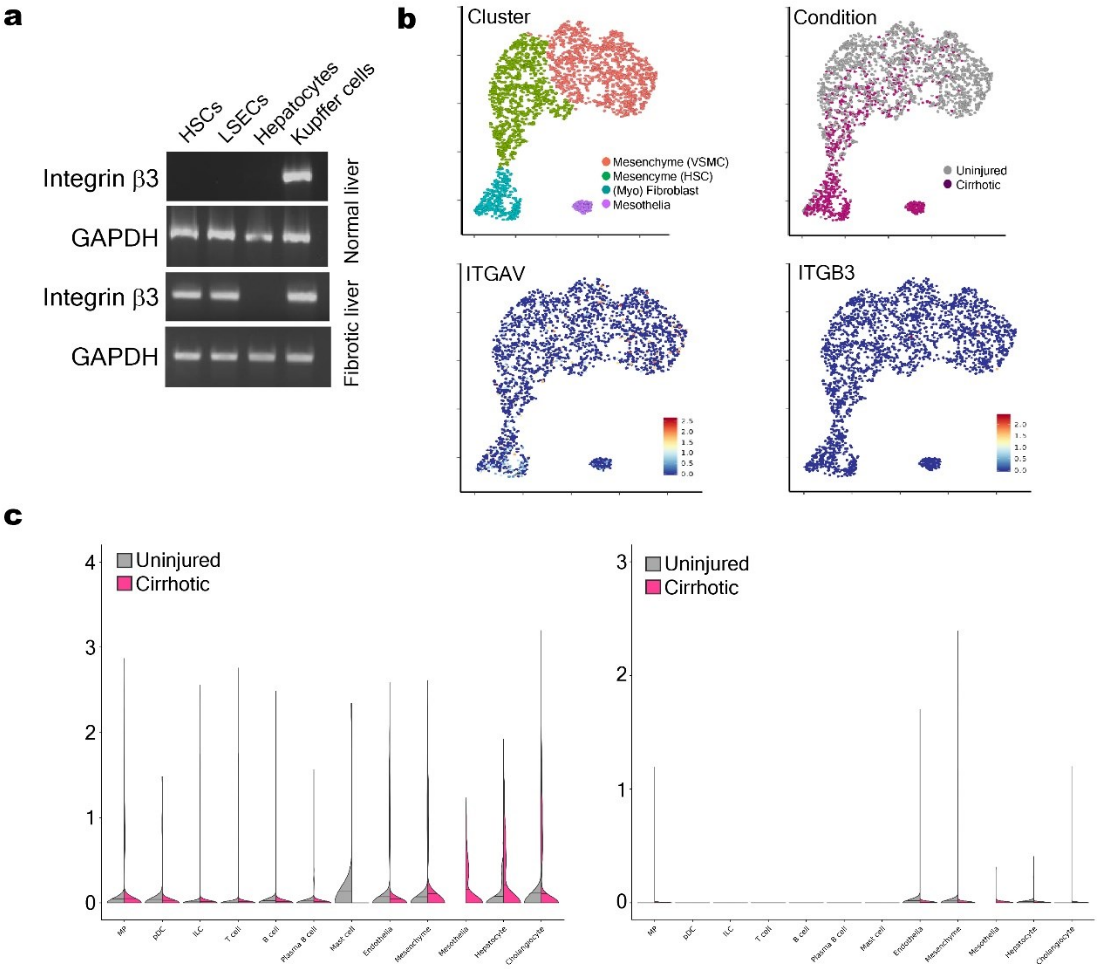

Supplementary Figure 1: Integrin αv and β3 expression profile in different cell types in healthy or cirrhotic liver.

(A) Total RNA was extracted from different cell populations from normal healthy or fibrotic livers and. Levels of mRNA of integrin β3 was determined by RT-PCR. The mRNA levels of GAPDH were probed as a control. **(B)** Clusters of cells were annotated according to disease condition (healthy and Cirrhotic livers; Top Panels) and gene expression for integrin αV and β3 (Bottom panels) was assessed in these clusters. **(C)** Gene expression profiles (Integrin αV: Left panel, Integrin β3: Right panel) for different cell populations were assessed in both normal healthy (Uninjured) or cirrhotic livers.

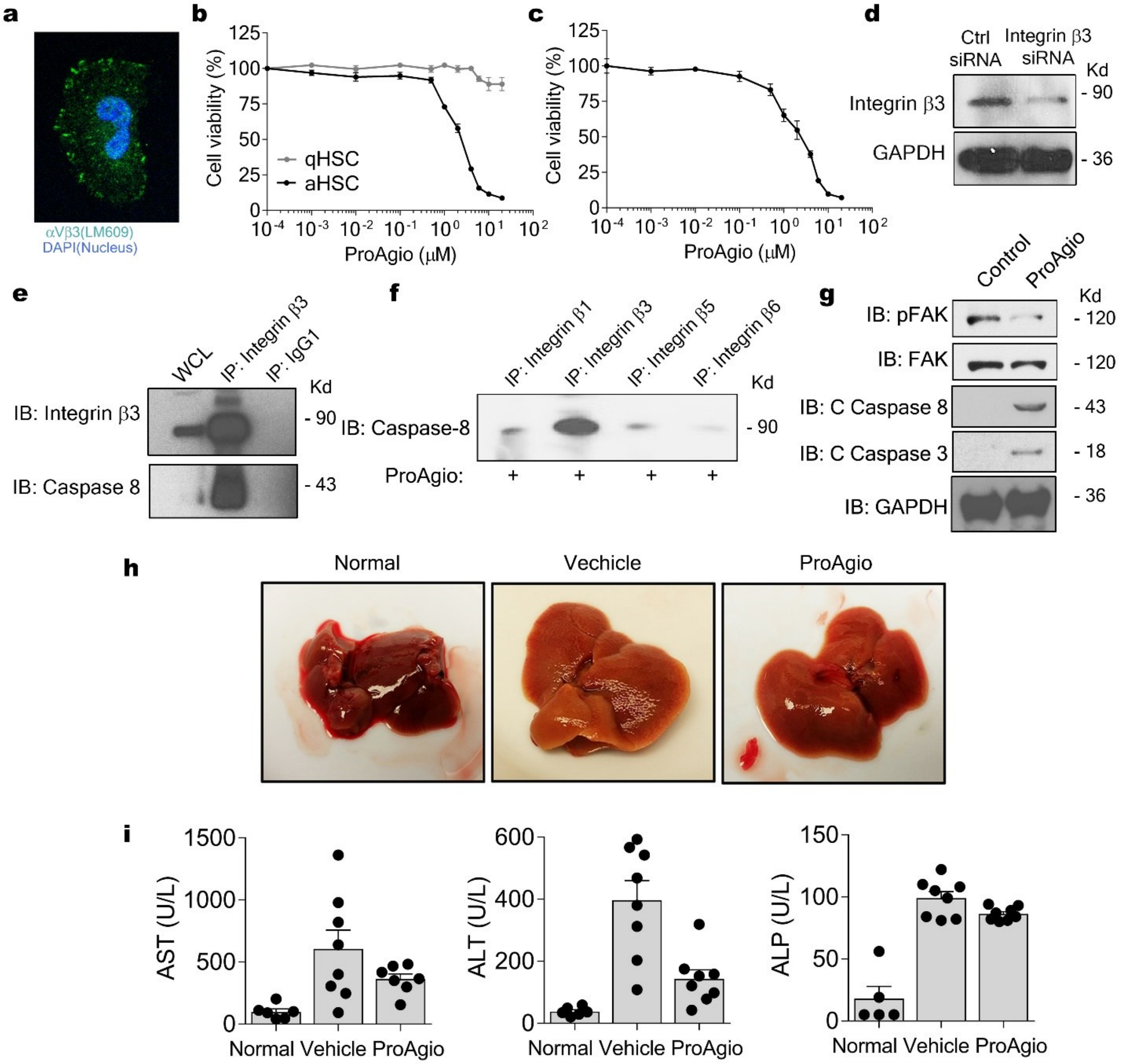

a
αVβ3(LM609)
DAPI(Nucleus)
b
Cell viability (%)
qHSC
aHSC
ProAgio (μM)
c
Cell viability (%)
ProAgio (μM)
d
Ctrl siRNA
Integrin β3 siRNA
Kd
Integrin β3
GAPDH
- 90
- 36
e
WCL
IP: Integrin β3
IP: IgG1
Kd
IB: Integrin β3
IB: Caspase 8
- 90
- 43
f
IP: Integrin β1
IP: Integrin β3
IP: Integrin β5
IP: Integrin β6
Kd
IB: Caspase-8
- 90
ProAgio: + + + +
g
Control
ProAgio
Kd
IB: pFAK - 120
IB: FAK - 120
IB: C Caspase 8 - 43
IB: C Caspase 3 - 18
IB: GAPDH - 36
h
Normal
Vechicle
ProAgio
i
AST (U/L)
ALT (U/L)
ALP (U/L)
Normal Vehicle ProAgio

Supplementary Figure 2: ProAgio reverses liver fibrosis

(A) Representative images of immunofluorescence staining of LX-2 cells using anti-integrin $\alpha_v\beta_3$ antibody LM609 (green). Blue is DAPI stains. **(B, C)** Cell viability of primary human HSC (B) and LX-2 cells (C) that were treated by indicated concentrations of ProAgio was measured by MTT assay. Primary human HSCs were cultured in day 1 without TGF-β (inactivated, qHSC) or seven days in presence of 5 ng/ml TGF-β (activated, aHSCs) before ProAgio treatment. **(D)** Expression of integrin β3 in LX-2 cells with (Integrin β3 siRNA) or without (Control siRNA) β3 knockdown was analyzed by immunoblot (IB: Integrin β3). **(E)** ProAgio treated LX-2 cells were subjected to immunoprecipitation analysis of integrin β3 (IP: Integrin β3) and were immunoblotted for Caspase 8 (IB: Caspase 8) with IgG as control. **(F)** ProAgio treated LX-2 cells were subjected to immunoprecipitation analysis of several integrins β1, β3, β5, and β6 (IP: Integrin β1, β3, β5, and β6) and immunoblotted for ProAgio (IB: ProAgio). **(G)** Immunoblot analyses of FAK (IB: FAK), phosphorylated FAK (IB: pFAK), cleaved caspase 8, (IB: C Caspase 8), and cleaved caspase 3 (IB: C Caspase 3) levels in extracts of LX2 cells under indicated treatment. Immunoblot of GAPDH (IB: GAPDH) in (D) and (G) is a loading control. **(H)** Representative images of livers harvested from mice treated with indicated agents. **(I)** Levels of serum markers in blood circulation of animals that were treated by the indicated agents were analyzed via commercial service (CPath). Error bars in (B) and (C) are standard deviations of five independent experiments. Error bars in (I) are standard deviations of calculation of six randomly selected mice from each treatment group. Normal in (H) and (I) are control mice without the fibrosis induction and treatment.

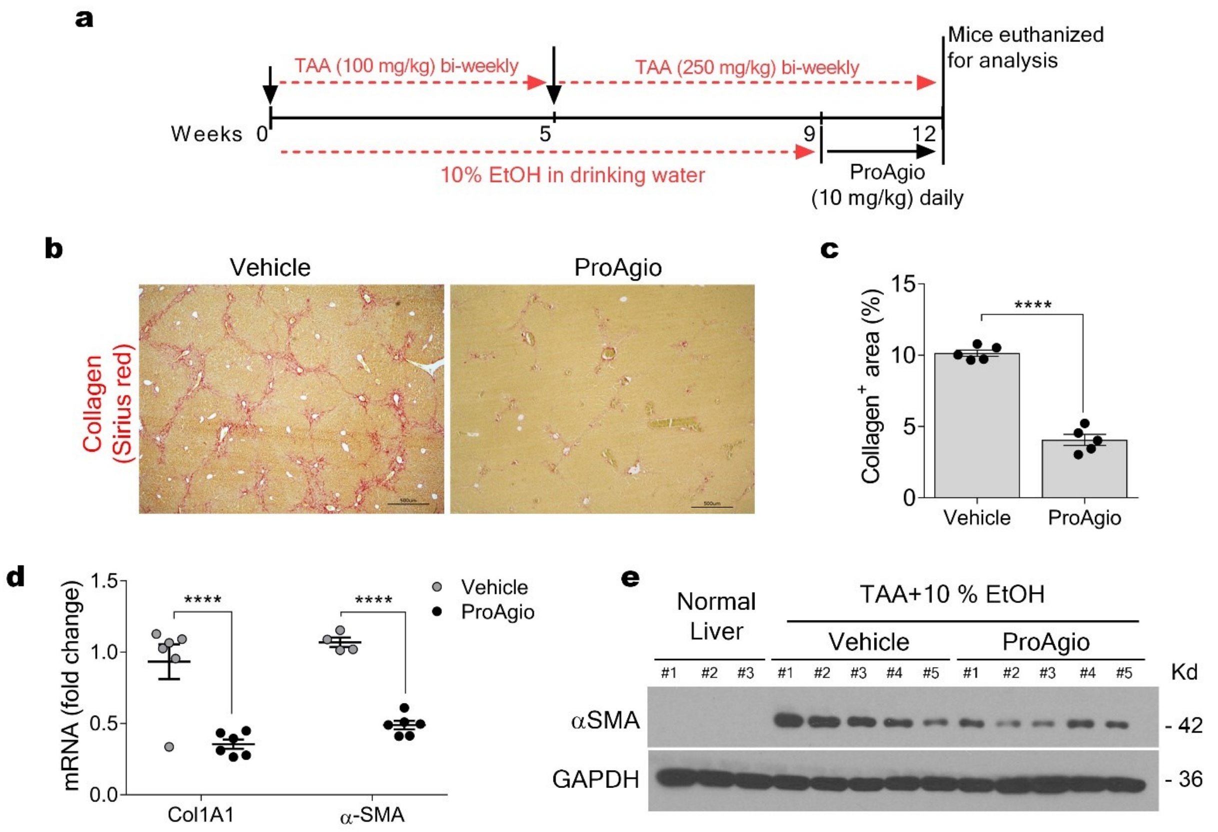

Supplementary Figure 3: ProAgio reverse liver fibrosis under continuous fibrosis induction condition.

(**A**) Schematic illustration of the schedule of TAA/alcohol liver fibrosis induction and ProAgio treatments (red arrows). Note TAA fibrosis induction continues during ProAgio treatment. **(B)** Representative images of Sirius red stain of sections of liver tissues from mice treated with indicated agents. **(C)** Quantitation of collagen levels in Sirius red stain of sections of liver tissues from mice treated with indicated agents using ImageJ software. **(D)** RT-PCR analyses of α-SMA and collagen 1 (Col1A1) mRNA levels in liver extracts of animals under indicated treatment. The mRNA levels are presented as fold changes comparing to the control (non-fibrotic and untreated animals) as reference. **(E)** Immunoblot analyses of α-SMA (α-SMA) levels in liver extracts of individual mouse (numbered on top of picture) under indicated treatment. Immunoblot of GAPDH (GAPDH) is a loading control. Error bars in (C) are standard deviations of measurements of 10 mice. Error bars in (D) are standard deviations of measurements of 5 mice. Normal liver in (E) are the control mice without the fibrosis induction and treatment.

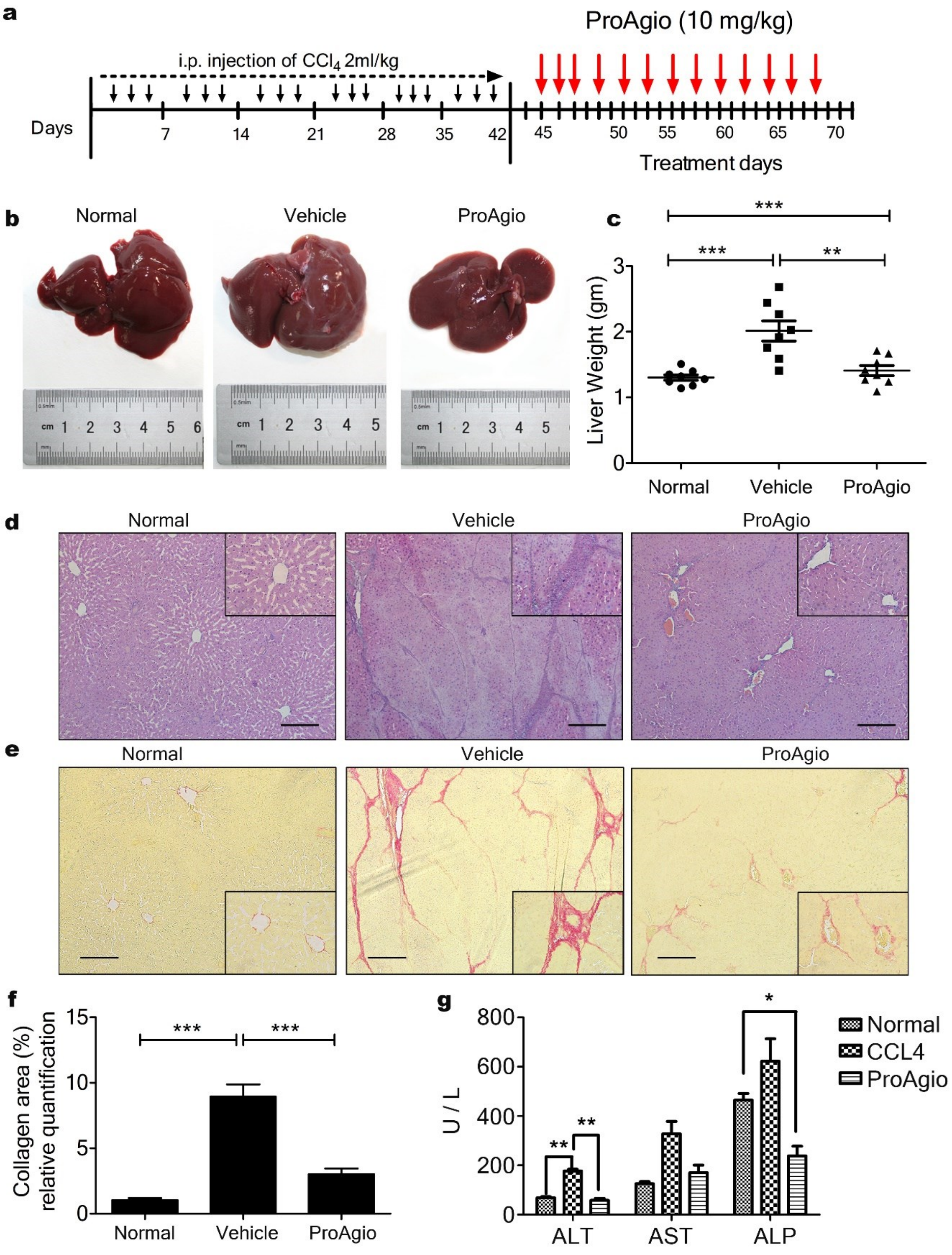
a
ProAgio (10 mg/kg)
i.p. injection of CCl4 2ml/kg
Days
7
14
21
28
35
42
45
50
55
60
65
70
Treatment days
b
Normal
Vehicle
ProAgio
c
Liver Weight (gm)
Normal
Vehicle
ProAgio
d
Normal
Vehicle
ProAgio
e
Normal
Vehicle
ProAgio
f
Collagen area (%) relative quantification
Normal
Vehicle
ProAgio
g
U / L
ALT
AST
ALP
Normal
CCL4
ProAgio

Supplementary Figure 4: ProAgio reverses CCl_4 induced liver fibrosis.
(A) Schematic illustration of the schedule of CCl_4 liver fibrosis induction and subsequent ProAgio treatments (red arrows). **(B)** Collected liver and **(C)** liver weights of the mice treated by indicated agents at the end point of experiments. Representative images of H&E **(D)** and Sirius red **(E)** stain of liver tissue sections from mice treated with indicated agents. **(F)** Quantitation of collagen levels in Sirius red stains using ImageJ software. Quantification was calculated from measurements of 10 mice. Four randomly selected tissue sections per animal and three randomly selected view fields in each section were quantified. The quantity of collagen levels in Sirius red stain is presented as % of total area. **(G)** Levels of serum markers in blood circulation of animals that were treated by the indicated agents were analyzed via commercial service (CPath). Error bars in (F) and (G) are standard deviations of calculation of six randomly selected mice from each treatment group. Normal in (B), (C), (D), (E), (F), and (G) are the mice without fibrosis induction and treatment.

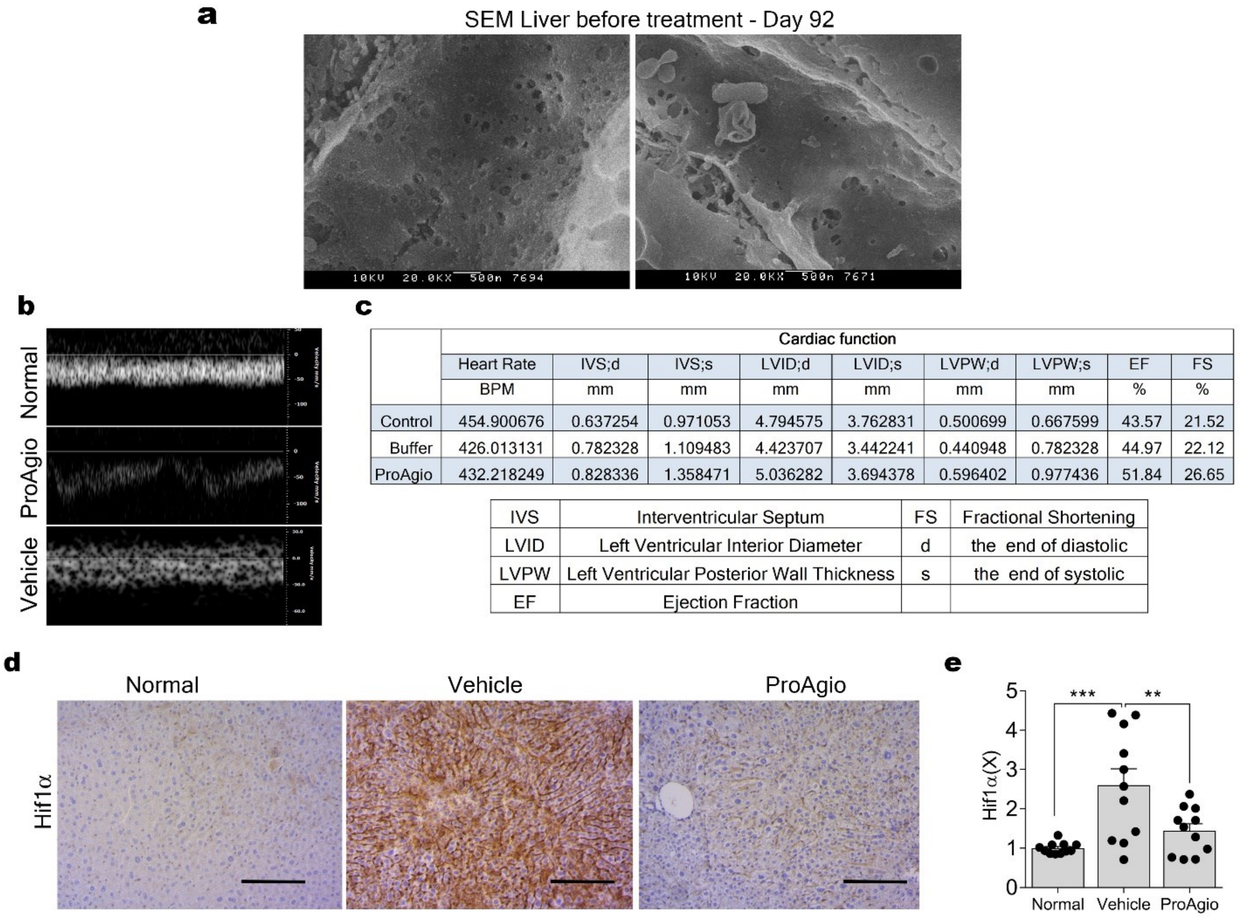

	Cardiac function								
	Heart Rate	IVS;d	IVS;s	LVID;d	LVID;s	LVPW;d	LVPW;s	EF	FS
	BPM	mm	mm	mm	mm	mm	mm	%	%
Control	454.900676	0.637254	0.971053	4.794575	3.762831	0.500699	0.667599	43.57	21.52
Buffer	426.013131	0.782328	1.109483	4.423707	3.442241	0.440948	0.782328	44.97	22.12
ProAgio	432.218249	0.828336	1.358471	5.036282	3.694378	0.596402	0.977436	51.84	26.65

IVS	Interventricular Septum	FS	Fractional Shortening
LVID	Left Ventricular Interior Diameter	d	the end of diastolic
LVPW	Left Ventricular Posterior Wall Thickness	s	the end of systolic
EF	Ejection Fraction		

Supplementary Figure 5: (A) Representative SEM images of liver sections from mice before fibrosis induction and 92 days after TAA/alcohol fibrosis induction. **(B)** Representative Doppler images showing waveforms of Doppler blood velocity in the hepatic portal vein of mice treated by the indicated agents. **(C)** Cardiac outputs of the Doppler imaging mice that treated by indicated agents. **(D, E)** Representative images of IHC stain of Hif1α (D) and quantitation (E) of Hif1α IHC positive stain area of liver tissue sections from mice treated with indicated agents. The quantity of Hif1α IHC is presented as fold changes in Hif1α positive stain area compared to that of non-fibrotic mice (normal). Quantifications of Hif1α IHC positive stain areas were calculated from measurements of 10 mice. Four randomly selected tissue sections per animal and three randomly selected view fields in each section were quantified. Normal in (B), (D), and (E) and control in (C) are the control mice without the fibrosis induction and treatment. Error bars in (E) are standard deviations of measurements of 10 mice.

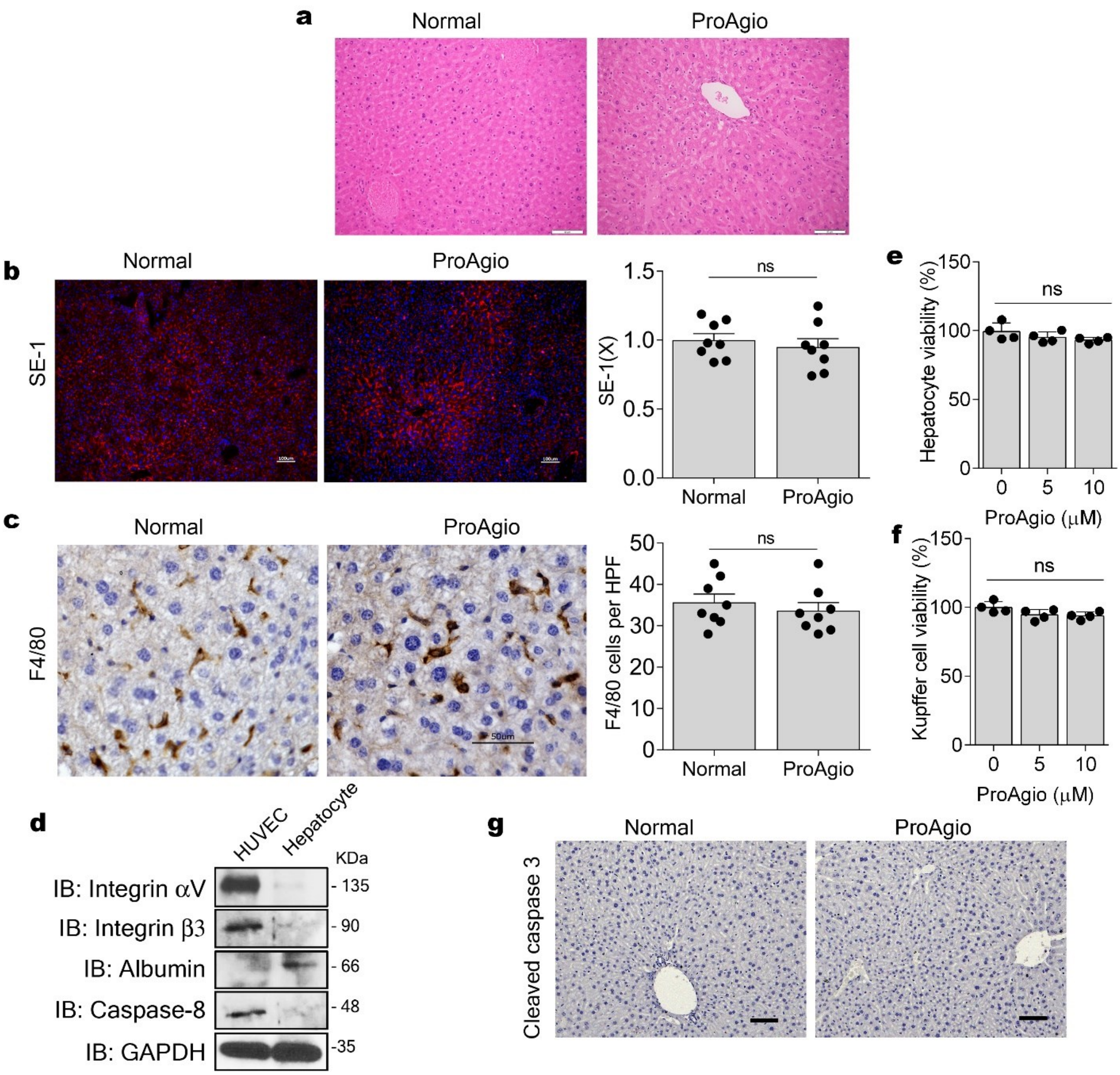
a
Normal
ProAgio
b
Normal
ProAgio
SE-1
SE-1(X)
1.5
1.0
0.5
0.0
ns
Normal
ProAgio
e
Hepatocyte viability (%)
150
100
50
0
ns
0
5
10
ProAgio (μM)
c
Normal
ProAgio
F4/80
F4/80 cells per HPF
50
40
30
20
10
0
ns
Normal
ProAgio
f
Kupffer cell viability (%)
150
100
50
0
ns
0
5
10
ProAgio (μM)
d
HUVEC
Hepatocyte
KDa
IB: Integrin αV
- 135
IB: Integrin β3
- 90
IB: Albumin
- 66
IB: Caspase-8
- 48
IB: GAPDH
-35
g
Normal
ProAgio
Cleaved caspase 3

Supplementary Figure 6: (A) Represent images of hematoxylin staining of tissue sections of liver collected from the mice that were treated with indicated agents. **(B) (Left)** Represent images and **(Right)** quantitation of IF staining of tissue sections of liver collected from the mice that were treated with indicated agents using anti- mouse/SE-1 antibody, recognizing LSEC specific marker. Quantitation of SE-1 stains is presented as fold changes compared to that of buffer treated mice. **(C) (Left)** Represent images and **(Right)** quantitation of IHC staining of tissue sections of liver collected from the mice that were treated with indicated agents using anti-mouse/F4/80 antibody, recognizing Kupffer cells and macrophages specific marker. Quantitation of F4/80 stains is presented as average number of F4/80 positive stain cells per view field. **(D)** Expression of integrin αv and β3 in primary human hepatocyte cells and HUVEC was analyzed by immunoblot of cell extracts using ant-integrin αv (IB:Integrin αv) and β3 (IB:Integrin β3) antibodies. Immunoblot analyses of caspase 8 (IB:Caspase 8) indicate induction of apoptosis by ProAgio. Immunoblot of albumin (IB:Albumin) and GAPDH (IB:GAPDH) are loading control. **(E)** and **(F)** Effects of ProAgio at indicated concentrations on viability of human primary hepatocyte cells (E) and kupffer cells (F) were analyzed by MTT assay. Cell viability is presented as average of % of viable cells in each view field. **(G)** Representative images of IHC staining of cleaved caspase 3 in sections of liver of normal mice (without fibrosis induction) treated with vehicle (normal) or ProAgio (20 mg/kg, i.p. 10 doses). Error bars in (B) and (C) are standard deviations of calculation of ten treatment mice from each treatment group. Error bars in (E) and (F) are standard deviations of five independent experiments. The images are typical of stains of at least two view fields of four sections from each animal.

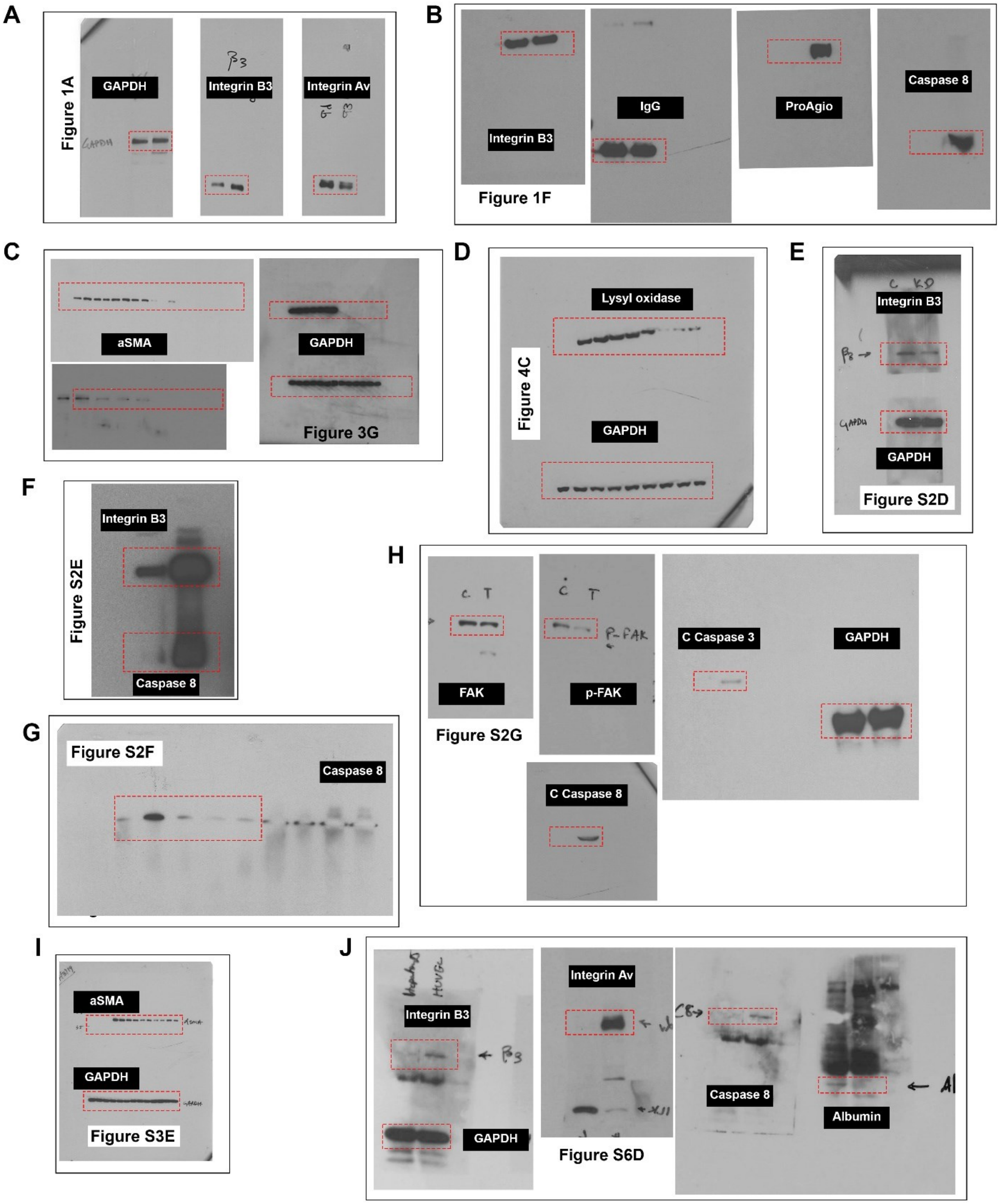

Supplementary Figure 7: Full scanned uncropped western blots
