# Supplementary material for: Targeting integrin αvβ3 by a rationally designed protein for chronic liver disease treatment
Source: Commun Biol. 2021 Sep 16;4:1087. doi: 10.1038/s42003-021-02611-2 (PMC8445973; doi:10.1038/s42003-021-02611-2)
Supplement: Supplementary file 3 — Description of Additional Supplementary Files [file 42003_2021_2611_MOESM3_ESM.pdf]

Description of Additional Supplementary Files

File name: Supplementary Data 1.

Description: Source data for plots and graphs.
