# Supplementary material for: Targeting integrin αvβ3 by a rationally designed protein for chronic liver disease treatment
Source: Commun Biol. 2021 Sep 16;4:1087. doi: 10.1038/s42003-021-02611-2 (PMC8445973; doi:10.1038/s42003-021-02611-2)
Supplement: Supplementary file 5 — Reporting Summary [file 42003_2021_2611_MOESM5_ESM.pdf]

nature research

Corresponding author(s): Zhi-Ren Liu

Last updated by author(s): Jun 22, 2021

Reporting Summary

Nature Research wishes to improve the reproducibility of the work that we publish. This form provides structure for consistency and transparency in reporting. For further information on Nature Research policies, see our Editorial Policies and the Editorial Policy Checklist.

Statistics

For all statistical analyses, confirm that the following items are present in the figure legend, table legend, main text, or Methods section.

n/a	Confirmed	
☐	☒	The exact sample size (*n*) for each experimental group/condition, given as a discrete number and unit of measurement
☐	☒	A statement on whether measurements were taken from distinct samples or whether the same sample was measured repeatedly
☐	☒	The statistical test(s) used AND whether they are one- or two-sided<br>*Only common tests should be described solely by name; describe more complex techniques in the Methods section.*
☒	☐	A description of all covariates tested
☒	☐	A description of any assumptions or corrections, such as tests of normality and adjustment for multiple comparisons
☐	☒	A full description of the statistical parameters including central tendency (e.g. means) or other basic estimates (e.g. regression coefficient) AND variation (e.g. standard deviation) or associated estimates of uncertainty (e.g. confidence intervals)
☒	☐	For null hypothesis testing, the test statistic (e.g. *F*, *t*, *r*) with confidence intervals, effect sizes, degrees of freedom and *P* value noted<br>*Give P values as exact values whenever suitable.*
☒	☐	For Bayesian analysis, information on the choice of priors and Markov chain Monte Carlo settings
☒	☐	For hierarchical and complex designs, identification of the appropriate level for tests and full reporting of outcomes
☒	☐	Estimates of effect sizes (e.g. Cohen's *d*, Pearson's *r*), indicating how they were calculated

Our web collection on statistics for biologists contains articles on many of the points above.

Software and code

Policy information about availability of computer code

Data collection	N/A
Data analysis	N/A

For manuscripts utilizing custom algorithms or software that are central to the research but not yet described in published literature, software must be made available to editors and reviewers. We strongly encourage code deposition in a community repository (e.g. GitHub). See the Nature Research guidelines for submitting code & software for further information.

Data

Policy information about availability of data

All manuscripts must include a data availability statement. This statement should provide the following information, where applicable:

- Accession codes, unique identifiers, or web links for publicly available datasets
- A list of figures that have associated raw data
- A description of any restrictions on data availability

All of the figures with their associated raw data are found in Supplementary Figure 7

Field-specific reporting

Life sciences study design

All studies must disclose on these points even when the disclosure is negative.

Sample size	Sample sizes were determined to ensure sufficient statistical differences
Data exclusions	N/A
Replication	Three in vivo experiments were performed with the n number mentioned in the figure legends<br>In vitro experiments were repeated at a minimum of three times to ensure consistent results
Randomization	All animals were separated into groups based on their treatment regimens and randomly chosen for tissue analyses.
Blinding	All the authors were not involved in a blind study throughout the course of the study.

Reporting for specific materials, systems and methods

We require information from authors about some types of materials, experimental systems and methods used in many studies. Here, indicate whether each material, system or method listed is relevant to your study. If you are not sure if a list item applies to your research, read the appropriate section before selecting a response.

Materials & experimental systems

n/a	Involved in the study	
☐	☒	Antibodies
☐	☒	Eukaryotic cell lines
☒	☐	Palaeontology and archaeology
☐	☒	Animals and other organisms
☒	☐	Human research participants
☒	☐	Clinical data
☒	☐	Dual use research of concern

Methods

n/a	Involved in the study	
☒	☐	ChIP-seq
☒	☐	Flow cytometry
☒	☐	MRI-based neuroimaging

Antibodies

Antibodies used	α-SMA Sigma A2547<br>cleaved caspase-8 Cell Signaling Technology 9496<br>PECAM1 Abcam Ab32457<br>SE-1 Novus Biologicals NB11068095<br>GAPDH Santa-Cruz Biotechnology Sc-47724<br>Hif1α Millipore MAB5382<br>Integrin αV Cell Signaling Technology 4711<br>Integrin β3 Millipore AB2984
Validation	All antibodies obtained were previously validated by the vendors and published.

Eukaryotic cell lines

Policy information about cell lines

Cell line source(s)	Hepatic stellate cells - Sciencell Online (5300)<br>Creative bioarray #CSC-C1496<br>Human hepatocytes - Lonza (HUCPI)<br>Human Kupffer cells - ThermoFisher (HUKCCS)<br>LX-2 Human hepatic stellate cells - Millipore (SCC064)
Authentication	All cell lines were authenticated by the vendor.
Mycoplasma contamination	All cell lines were confirmed to be tested negative for mycoplasma by the vendor.
Commonly misidentified lines<br>(See ICLAC register)	N/A

April 2020

Animals and other organisms

Policy information about studies involving animals; ARRIVE guidelines recommended for reporting animal research

Laboratory animals	BALB/c, C57BL6, aged 7-8 weeks
Wild animals	N/A
Field-collected samples	N/A
Ethics oversight	All experiments were performed under approved IACUC protocols

Note that full information on the approval of the study protocol must also be provided in the manuscript.
